# Ketogenic Diet Effects on Liver Health in Metabolic Dysfunction–Associated Steatotic Liver Disease: A Systematic Review

**DOI:** 10.1111/liv.70942

**Published:** 2026-11-13

**Authors:** Patrizia Palumbo, Harini Krishnapillai, Fleur de Vreeze, Elena Buzzetti, Ger H. Koek

**Affiliations:** ^1^ School of Nutrition and Translational Research in Metabolism Maastricht University Maastricht the Netherlands; ^2^ Division of Metabolic Diseases and Clinical Nutrition University Hospital of Modena Modena Italy; ^3^ Faculty of Health, Medicine and Life Sciences Maastricht University Maastricht the Netherlands; ^4^ Internal Medicine and Centre for Hemochromatosis and Hereditary Liver Diseases, ERN‐EuroBloodNet Center for Iron Disorders, AOU Policlinico University of Modena and Reggio Emilia Modena Italy; ^5^ MUMC+, Department of Gastroenterology and Hepatology Maastricht University Medical Center Maastricht the Netherlands

**Keywords:** carbohydrate‐restricted diet, diet therapy, fatty liver, ketogenic diet

## Abstract

Metabolic dysfunction–associated steatotic liver disease (MASLD) is the most prevalent chronic liver condition and is linked to unhealthy lifestyle patterns. Dietary modification remains the cornerstone of management. Among available nutritional strategies, ketogenic diets (KDs) have gained increasing attention. This systematic review evaluated the impact of KD, versus no dietary intervention or control diets, on hepatic and metabolic outcomes in adults with MASLD. Following PRISMA guidelines, PubMed, Scopus and Web of Science were searched through June 2026. Two reviewers independently screened records, assessed eligibility and extracted data. Risk of bias was evaluated using CASP for randomized controlled trials (RCTs) and ROBINS‐I for non‐randomized studies. Meta‐analysis was not feasible due to study heterogeneity. Fourteen studies were included: five RCTs and nine prospective cohorts, involving 776 adults with MASLD and/or hepatic steatosis. KD was associated with improvements in MASLD severity indices, anthropometric measures, lipid profile, glycaemic markers and liver enzymes. Among RCTs, liver fat decreased by −4.77% (PDFF) to a 77% (relative MRI‐LFF), with reductions in body weight (up to −12.0 kg) and waist circumference (up to −9.6 cm). Prospective studies showed similar findings, including reductions in controlled attenuation parameter (up to −57 dB/m), intrahepatic triglyceride (−31%) and Fatty Liver Index (−14.03 points). Evidence regarding liver stiffness and fibrosis‐related outcomes was more limited and less consistent. KD may improve disease severity, hepatic steatosis, anthropometric measures, glycaemic control, lipid metabolism and liver‐related biochemical markers in adults with MASLD. However, methodological heterogeneity precludes outcomes assessment, and limited evidence on fibrosis‐related evidence precludes firm conclusions. Larger, longer‐standardized RCTs are needed to clarify KD's role in MASLD management.

## Key Points

- Ketogenic dietary interventions may reduce liver fat content in adults with metabolic dysfunction–associated steatotic liver disease, as shown by improvements in imaging‐based steatosis measures.
- Consistent reductions in body weight, waist circumference, fasting glucose, insulin resistance and triglycerides suggest broad metabolic benefits; however, these effects cannot be fully disentangled from the influence of caloric restriction and weight loss.
- Improvements in serum liver enzymes indicate a potential favourable effect on hepatocellular injury.
- Evidence remains limited by heterogeneous study designs, variable intervention protocols, differences in hepatic outcome assessment methods and relatively small sample sizes.
- Larger, rigorously designed randomized controlled trials are required to clarify the therapeutic role of ketogenic diets in the management of metabolic dysfunction–associated steatotic liver disease.

## Lay Summary

This systematic review suggests that ketogenic diets may reduce liver fat and improve body weight, blood sugar control, blood fat levels and liver blood tests in adults with metabolic dysfunction–associated steatotic liver disease. However, differences among the available studies and limited evidence regarding liver scarring mean that larger, longer and well‐designed studies are required before definitive conclusions can be reached.

**Abbreviations**
5:2 IF: 5:2 intermittent fasting
ALT: alanine aminotransferase
AST: aspartate aminotransferase
BMI: body mass index
BW: body weight
CAP: controlled attenuation parameter
CASP: critical appraisal skills programme
CI: confidence interval
CKD: chronic kidney disease
CMRF: cardiometabolic risk factor
CVD: cardiovascular disease
DASH: dietary approach to stop hypertension
ELF: enhanced liver fibrosis score
FGF: fibroblast growth factor
FIB‐4: fibrosis‐4 index
FM: fat mass
GGT: gamma‐glutamyl transferase
HbA1c: glycated haemoglobin
HDL‐c: high‐density lipoprotein cholesterol
HOMA‐IR: homeostatic model assessment of insulin resistance
IHTG: intrahepatic triglycerides
KD: ketogenic diet
LC: low‐carbohydrate diet
LCHF: low‐carbohydrate high‐fat diet
LDL‐c: low‐density lipoprotein cholesterol
MASH: metabolic dysfunction–associated steatohepatitis
MASLD: metabolic dysfunction–associated steatotic liver disease
MRE: magnetic resonance elastography
MRS: magnetic resonance spectroscopy
NAFLD: non‐alcoholic fatty liver disease
NI: no information
NITs: non‐invasive tests
OSAS: obstructive sleep apnoea syndrome
PCOS: polycystic ovary syndrome
PDFF: proton density fat fraction
PNPLA3: patatin‐like phospholipase domain‐containing protein 3
PRISMA: preferred reporting items for systematic reviews and meta‐analyses
RCT: randomized controlled trial
ROBINS‐I: risk of bias in non‐randomized studies of interventions
SoC: standard of care
T2DM: type 2 diabetes mellitus
TC: total cholesterol
TG: triglycerides
VLCD: very low‐carbohydrate diet
VLCKD: very low‐calorie ketogenic diet
WC: waist circumference

## 1 Introduction

Metabolic dysfunction–associated steatotic liver disease (MASLD), formerly known as non‐alcoholic fatty liver disease (NAFLD), has emerged as a leading global health concern affecting more than a third of the global population [1]. The prevalence is notably higher in individuals with obesity (50.7%) and Type 2 diabetes mellitus (65.33%) (T2DM), making it one of the leading causes of chronic liver disease [1, 2, 3, 4].

MASLD is defined by hepatic steatosis occurring in the presence of cardiometabolic risk factors, reflecting its underlying metabolic origin [5].

MASLD develops through a multifactorial multiple‐hit process driven by insulin resistance, lipotoxicity, oxidative stress and inflammation, with emerging contributions from the gut–liver axis [6, 7, 8]. These mechanisms drive hepatic injury and, in about one‐third of individuals with liver fat above 5%, may lead to progressive disease including fibrosis, cirrhosis and hepatocellular carcinoma [4, 9, 10].

MASLD is also associated with increased cardiovascular and metabolic risk, further contributing to overall clinical burden [10, 11, 12, 13, 14]. Although pharmacological options are expanding, including incretin‐based therapies, fibroblast growth factor (FGF) agonists and the recent approval of agents such as resmetirom (both in Europe and the United States) and semaglutide in the United States for MASH with fibrosis, lifestyle modification remains the cornerstone of MASLD management [15, 16, 17]. In particular, weight reduction represents the primary therapeutic goal; however, achieving and maintaining clinically meaningful weight loss remains a major challenge in clinical practice [13, 18, 19, 20].

Given these challenges, various dietary patterns have been proposed for MASLD to support weight loss and improve metabolic health, including the Mediterranean diet, DASH diet, low‐fat and low‐carbohydrate diets, intermittent fasting and vegetarian diets, with the ketogenic diet (KD) emerging as a promising approach. By restricting carbohydrate intake to < 50 g/day and increasing fat consumption, KDs induce nutritional ketosis and may reduce liver fat [21], improve stiffness, enhance insulin sensitivity [21, 22] and modulate the gut–liver axis [21, 23]. Multiple ketogenic variants exist (Table S1), although a unified classification is still lacking [24].

Despite these promising findings, several uncertainties still remain, particularly regarding long‐term efficacy, safety and individual variability in response. The existing literature is often difficult to interpret consistently, as studies differ in design, in how ketogenic diets are defined along with the way hepatic outcomes are assessed. In addition, much of the current evidence is derived from narrative reviews or studies that do not specifically focus on MASLD populations defined according to the most recent diagnostic criteria. As a result, the overall evidence base remains fragmented, and direct comparisons between ketogenic diets and no dietary intervention in MASLD are still limited. In this context, this review expands upon existing literature by providing a systematic synthesis of clinical evidence on ketogenic diets in MASLD. Compared with previous narrative reviews, it applies a structured methodology and formal risk‐of‐bias assessment and focuses specifically on studies using contemporary definitions of MASLD. By integrating data across multiple domains, this review offers a more comprehensive and standardized overview of the potential role of ketogenic diets in this population. Accordingly, the aim of this systematic review is to critically evaluate the effects of the KD, compared to no dietary intervention, on hepatic and metabolic outcomes in patients with MASLD.

## 2 Methods

The review was structured as proposed by the Preferred Reporting Items for Systematic Reviews and Meta‐Analyses (PRISMA) [25] guidelines. The completed checklist is provided as Supporting Information S1.

### 2.1 Eligibility Criteria

Articles were selected according to the PRISMA guidelines. Eligibility criteria were defined as follows: (1) article should be written in English; (2) studies involved patients diagnosed with MASLD. Studies using NAFLD definitions were also considered eligible when they were broadly consistent with MASLD criteria, based on the reported presence of metabolic risk factors and the exclusion of other causes of liver disease, where specified in the original studies; (3) studies investigating the effect of KD, including their variations, provided they meet the definition of a high‐fat, low‐carbohydrate diet inducing ketosis. In this review, ketogenic diets were generally defined as interventions involving a marked restriction in carbohydrate intake (typically below 50 g/day), or as very‐low‐calorie ketogenic diets (VLCKD) or very‐low‐energy ketogenic therapy (VLEKT) when described as such in the original studies. This definition was chosen to reflect the variability in dietary protocols across studies while ensuring inclusion of interventions capable of inducing nutritional ketosis; (4) both studies with and without a control group were included, (5) studies reporting at least one of the following outcomes: hepatic steatosis (e.g., CAP, PDFF, IHTG), liver enzyme levels or other liver‐related biochemical parameters, and/or markers of hepatic fibrosis assessed either through liver stiffness measurement (FibroScan or MRE), histology, or other validated non‐invasive tests (NITs) such as FIB‐4, NAFLD Fibrosis Score, ELF, or comparable indices; (6) no restrictions were made regarding publishing date and study design.

### 2.2 Information Sources and Search Strategy

The literature search was conducted in multiple databases, including PubMed, Scopus and Web of Science, and was last updated in June 2026. The search strategy was structured around three predefined conceptual domains: (1) the target condition, (2) the ketogenic dietary intervention and (3) the relevant clinical outcomes.

For the condition, we included controlled vocabulary terms (e.g., MeSH) and free‐text terms referring to metabolic dysfunction‐associated steatotic liver disease and related entities (e.g., ‘Fatty Liver’, ‘Non‐Alcoholic Fatty Liver Disease’, ‘MASLD’, ‘NAFLD’, ‘hepatic steatosis’).

For the intervention, we used the MeSH term ‘Diet, Ketogenic’ and associated keywords such as ‘ketogenic diet’, ‘very‐low‐calorie ketogenic diet (VLCKD)’, ‘low‐carbohydrate high‐fat diet’ (LCHF) and ‘very‐low‐carbohydrate diet’ (VLCD).

Outcome‐specific terms (e.g., liver steatosis, liver stiffness, liver enzymes, glycaemic or lipid markers) were not incorporated into the search string to avoid unnecessary restrictions but were applied during screening as part of the eligibility criteria.

The three conceptual domains were combined with AND to identify studies evaluating KD in MASLD/NAFLD populations. No restrictions on publication date, study design, or age were applied. Only human studies published in English were considered.

All records retrieved from the different databases were combined and duplicates were removed prior to screening. The full, expanded query is provided in Supporting Information S5.

### 2.3 Study Selection

Screening and study selection were performed independently by two reviewers using Rayyan software. The screening process followed a hierarchical approach. After removing reviews and irrelevant publication types, titles and abstracts were screened to exclude studies not involving the MASLD/NAFLD population or not assessing KD. Full texts were then evaluated according to eligibility criteria, requiring at least one primary outcome related to liver steatosis or liver‐related outcomes. Cohen's kappa was calculated to quantify inter‐rater agreement.

### 2.4 Data Collection Process

Data were extracted using a standardized Excel form. For all included studies, two reviewers independently extracted information on study characteristics (e.g., author, publication date), participant characteristics, statistical methods used, type of intervention and a summary of the main findings on liver steatosis and liver‐related outcomes. Extracted data were then combined into four summary tables (Tables 1 and S2a–S2c).

**TABLE 1:** Study characteristics of included trials and cohorts evaluating ketogenic or low‐carbohydrate diets in adults with MASLD.

Study (author, year)	Duration	Study design	Arms	Mean age (years)	Participants (*n*)	Population	Steatosis evaluation method	Dietary intervention	Characteristic of the type of the dietary intervention
Cunha et al. (2020) [26]	2 months	Open, randomized controlled, prospective pilot study	2 arms	40.3 ± 11.3	39	Brazilian adults, 46 randomized (8 males, 38 females); 39 completers	PDFF > 5.4% (Baseline: 26/39 had steatosis)	VLCKD vs. LCD	Randomizing patients 1:1 into two groups to follow either a very‐low‐calorie ketogenic diet (VLCKD) or a standard low‐calorie (LC) diet for weight loss for 2 months with regular clinical assessments by an endocrinologist. Lifestyle guidance (diet and exercise) by dietitians and physical trainers.
Holmer et al. (2021) [27]	12 weeks	Open label randomized controlled trial	3 arms	SoC: 56 ± 9 5:2 IF: 57 ± 10 LCHF: 56 ± 12	74	Sweden adults, 33 males, 41 females	Transient elastography CAP > 280 dB/m and MRE (Baseline: 74/74 had steatosis)	SoC vs. 5:2 IF vs. LCHF	This study compared a low‐carbohydrate high‐fat (LCHF) diet, a 5:2 intermittent fasting diet, and standard of care (SoC), with participants randomized equally. The 5:2 group followed two fasting days (500 kcal/day for women, 600 kcal/day for men) and five non‐fasting days (2000 kcal/day for women, 2400 kcal/day for men), while the LCHF group consumed 1600 kcal/day (women) or 1900 kcal/day (men) with 5%–10% carbohydrates, 50%–80% fat, and 15%–40% protein; SoC focused on balanced dietary guidance. All outcomes were reassessed at week 12.
Hansen et al. (2023) [28]	6 months +3‐month follow‐up	Open‐label randomized controlled trial	2 arms	56 ± 10	165	Danish adults with Type 2 diabetes; 58% women; 88% had NAFLD/MASLD	Liver biopsy (NAS score), liver histology (Baseline: 145/165 had steatosis)	LCHF vs. HCLF	Participants were randomized to either a calorie‐unrestricted low‐carbohydrate high‐fat (LCHF) diet (< 20 E% carbohydrate, ~60 E% fat, 20–25 E% protein) or a high‐carbohydrate low‐fat (HCLF) diet (~45 E% carbohydrate, ~30 E% fat). The intervention lasted 6 months followed by a 3‐month post‐intervention follow‐up. The LCHF diet resulted in greater reductions in HbA1c, fasting glucose, body weight, waist circumference, and HOMA‐IR compared with HCLF. However, no significant superiority was observed for histological NAFLD improvement (NAS response).
Chirapongsathorn et al. (2025) [29]	8 weeks	Open label randomized controlled trial	2 arms	Ketogenic: 37.4 ± 7.5 Control: 40.2 ± 7.6	22 (after dropouts)	Thai adults 16 females, 8 males	Transient elastography CAP > 200 dB/m (Baseline: 22/22 had steatosis)	Hypocaloric KD vs. DASH	This study compared a ketogenic diet with general lifestyle advice based on the DASH diet over an 8‐week period. Participants were randomized to receive either a home‐delivered ketogenic diet (1500 kcal/day, ~5% carbohydrates, 70% fat, 25% protein) or counselling to follow a DASH diet (1500 kcal/day, ~60% carbohydrates, 25% fat, 15% protein). Both groups met with dietitians at baseline, week 4, and week 8, and adherence was monitored through food diaries and photo submissions. The ketogenic group achieved mild nutritional ketosis and showed greater reductions in body weight (−6.1 kg vs. −2.1 kg), waist circumference, fat mass, and visceral fat area compared to the control group. Improvements were also observed in systolic blood pressure and triglycerides, while liver stiffness and steatosis did not differ significantly between groups.
Farrell et al. (2026) [30]	24 weeks (12‐week diet +12‐week maintenance)	Single‐centre unblinded randomized controlled trial	2 arms	49 (IQR 42–57)	25	Adults with histologically confirmed MASLD and BMI 27–35 kg/m^2^	MRI‐LFF, CAP, LSM, liver biopsy (Baseline: 25/25 had steatosis)	Ketogenic VLED vs. Mediterranean Diet	Participants were randomized to either a ketogenic VLED (3151 kJ/day; two meal replacements plus one very‐low‐carbohydrate meal daily) or a Mediterranean diet (8950 kJ/day). Following the 12‐week intervention, the VLED group received low‐dose semaglutide (0.5 mg/week) for weight maintenance through week 24. Outcomes included MRI‐LFF, FibroScan parameters, liver histology, anthropometry and metabolic markers.
Tendler et al. (2007) [31]	6 months	Prospective, clinical pilot trial	Single arm	35.6 (range 24–50)	5	American adults, 2 males 3 females	Biopsy‐confirmed steatosis (Baseline: 5/5 had steatosis)	LCKD with commercial meal replacements	Participants followed a low‐carbohydrate ketogenic diet (LCKD) with < 20 g of carbohydrates per day, allowing unlimited meats, eggs, and limited cheese, salad, and low‐carb vegetables, with no calorie restrictions. They received multivitamins and supplements, attended group sessions biweekly for 3 months, then monthly.
Luukkonen et al. (2020) [22]	6 days	Prospective	Single arm	58.2 ± 2.8	10	Finnish adults, 5 males, 5 females	H‐MRS/MRE (Baseline: 10/10 had steatosis)	Hypocaloric KD	Patients followed a 6‐day hypocaloric ketogenic diet (1440 kcal/day) with 6% carbohydrates (≤ 25 g/day), 64% fat, and 28% protein. Meals were pre‐prepared and frozen, with adherence monitored via phone calls and a 3‐day food diary.
D'Abbondanza et al. (2020) [32]	25 days	Prospective observational study	Single arm	48 (IQR 20–67)	70	Italian adults with severe obesity and associated NAFLD; 42 females and 28 males	Abdominal ultrasonography (Baseline: 68/70 had steatosis)	VLCKD	Participants followed a very low‐carbohydrate ketogenic diet providing < 800 kcal/day and < 50 g/day carbohydrates, with protein intake of 1.4 g/kg ideal body weight. Ketosis was monitored through daily urinary ketone testing. Anthropometric, metabolic and liver‐related parameters were evaluated before and after intervention.
De Nucci et al. (2023) [33]	8 weeks	Real‐life Prospective	Single arm	42.1 ± 12.9	87	Italian adults, 29 males, 58 females	CAP > 215 dB/m (Baseline: 77/87 had steatosis)	VLCKD	Patients followed a VLCKD with daily intake limited to 800 kcal, including 20–50 g carbohydrates, 1–1.4 g/kg protein, and 15–30 g fat. Micronutrient supplements were provided to prevent deficiencies. In the first phase, meal replacements and vegetables were allowed, while a protein dish was added during the second phase. Participants were monitored at baseline (T0), 3 weeks (T1) and 8 weeks (T2).
Rinaldi et al. (2023) [34]	8 weeks	Real‐life Prospective	Single arm	40.2 ± 14.9	33	Italian adults, 9 males, 24 females	CAP > 302 dB/m	VLCKD	Participants followed an 8‐week Very Low‐Calorie Ketogenic Diet (VLCKD) with 20–50 g of carbohydrates, 1–1.4 g of protein per kg of ideal body weight, and 15–30 g of fat per day. The diet included meal replacements, at least 2 L of water daily, and micronutrient supplements to prevent deficiencies.
Sila et al. (2024) [35]	8 weeks	Real‐life Prospective	Single arm	41.9 ± 12.7	111	Italian adults, 36 males, 75 females	CAP > 275 dB/m (Baseline: 111/111 had steatosis)	VLCKD	Participants followed Very Low‐Calorie Ketogenic Diet (VLCKD) designed to induce ketosis through low carbohydrates and high fats, with significant caloric restriction to promote weight loss and metabolic improvements. They received regular monitoring, at T0, T1 (3 weeks) and T2 (8 weeks).
Haley et al. (2025) [36]	2 weeks	Single‐centre, longitudinal cohort study with intervention (open‐label)	2 arms	Overall: 54.0 ± 8.8 Low CAL: 52.3 ± 7.7 Low CHO: 55.7 ± 9.6	44	American adults, 8 males, 36 females	MRI‐based Proton Density Fat Fraction (PDFF ≥ 5%) *(Baseline: 30/44 had MASLD)*	Low carbohydrate (≤ 30 g/day) vs. Low calorie (~1200 kcal/day for women; ~1500 kcal/day for men)	This study compared two dietary approaches—a low‐carbohydrate diet and a low‐calorie diet—implemented over a two‐week period. Participants were randomized in blocks of five to receive either a carbohydrate‐restricted plan (≤ 30 g/day) or a calorie‐restricted plan (~1200 kcal/day for women and ~1500 kcal/day for men). All meals were prepared and delivered by a commercial service (Snap Kitchen) twice weekly to ensure adherence. Both interventions aimed to reduce hepatic triglyceride content. The low‐carbohydrate group consumed meals with very limited carbohydrates, moderate protein, and higher fat, while the low‐calorie group followed a balanced macronutrient distribution within the prescribed energy limits. Outcomes were assessed pre‐ and post‐intervention using MRI‐based proton density fat fraction (PDFF) and brain spectroscopy, revealing significant reductions in liver fat (mean difference = 3.1%), as well as decreases in cerebral glutamate and myo‐inositol concentrations.
Saslow et al. (2025) [37]	4 months	Single‐arm pilot trial	Single arm	55.5 ± 11.9	11	American adults 8 males and 3 females	MRI for liver fat and fibrosis	VLC diet	This study evaluated a web‐based very low–carbohydrate (VLC) program over 4 months in adults with MASLD and PNPLA3 risk genotype. Participants were taught to follow an ad libitum VLC eating pattern (20–35 g net carbohydrates per day), supported by weekly educational emails with videos and handouts, near‐daily text reminders, and personalized coaching. The program also incorporated behavioural strategies such as positive affect skills, mindful eating, goal setting, and self‐monitoring. Participants received cookbooks, a connected scale for weight tracking, and urine ketone strips. Physical activity (≥ 150 min/week) and adequate sleep were encouraged starting at week 6. Outcomes were assessed at baseline and after 4 months using MRI for liver fat and fibrosis, blood tests, and questionnaires. The intervention resulted in substantial reductions in liver fat (mean −33.2%, up to −53.1% in adherent participants), improvements in weight (≥ 5% loss in all participants), and favourable changes in metabolic markers, with high satisfaction and no serious adverse events reported.
Barrea et al. (2025) [38]	45 ± 2 days	Prospective outpatient study	Single arm	39.25 ± 4.89	80	Italian women with obesity and MASLD (FLI ≥ 60)	Fatty Liver Index (FLI) (Baseline: 80/80 had steatosis)	VLEKT	Participants followed the ketogenic phase of a Very Low Energy Ketogenic Therapy (VLEKT) providing < 800 kcal/day, < 30 g carbohydrates/day (13% CHO, 43% protein, 44% fat). Ketosis was monitored weekly through capillary β‐hydroxybutyrate measurements. Significant improvements were observed in anthropometric parameters, FLI, lipid profile, fasting glucose, liver enzymes and hs‐CRP.

### 2.5 Quality Assessment

Risk of bias was assessed independently by two reviewers. Randomized controlled trials (RCTs) were evaluated using the Critical Appraisal Skills Programme (CASP) tool [39], covering (1) study design, (2) methodology, (3) results and (4) applicability. The questions in the quality assessment form were answered by yes, no, or can't tell. Non‐randomized studies were evaluated using the ROBINS‐I tool [40], assessing (1) confounding, (2) selection of participants, (3) classification of interventions, (4) deviations from intended interventions, (5) missing data, (6) measurement of outcomes, (7) selection of the reported result and (8) overall bias. The items in the quality assessment were rated as critical, serious, moderate, low and no information (NI). A condensed version of the quality assessment is presented in Tables S3 and S4.

### 2.6 Summary Measures

Results extracted from the included studies are presented using descriptive statistics, such as percentages, means with standard deviations, or medians with interquartile ranges, depending on the nature of the data reported.

However, meta‐analysis was not feasible due to substantial heterogeneity in study designs (randomized and non‐randomized studies), ketogenic diet protocols, intervention duration and outcome measures used to assess hepatic and metabolic parameters. In addition, important inconsistencies in statistical reporting, particularly the limited and non‐uniform reporting of confidence intervals and other measures of uncertainty, further limited comparability across studies, precluding robust and meaningful effect size pooling or the construction of forest plots.

## 3 Results

### 3.1 Study Selection

The database search identified 696 articles across PubMed, Scopus and Web of Science. After removal of 529 duplicates, 167 records were screened by title and abstract, 153 articles were excluded and 14 underwent full text screening. The main reasons for exclusion were lack of MASLD diagnosis, use of non‐ketogenic dietary interventions, or absence of relevant outcomes. Ultimately, 14 studies met the inclusion criteria and were included in the final analysis (Figure 1). The calculated Cohen's kappa coefficient was 0.6567, indicating substantial agreement between reviewers.

**FIGURE 1:**
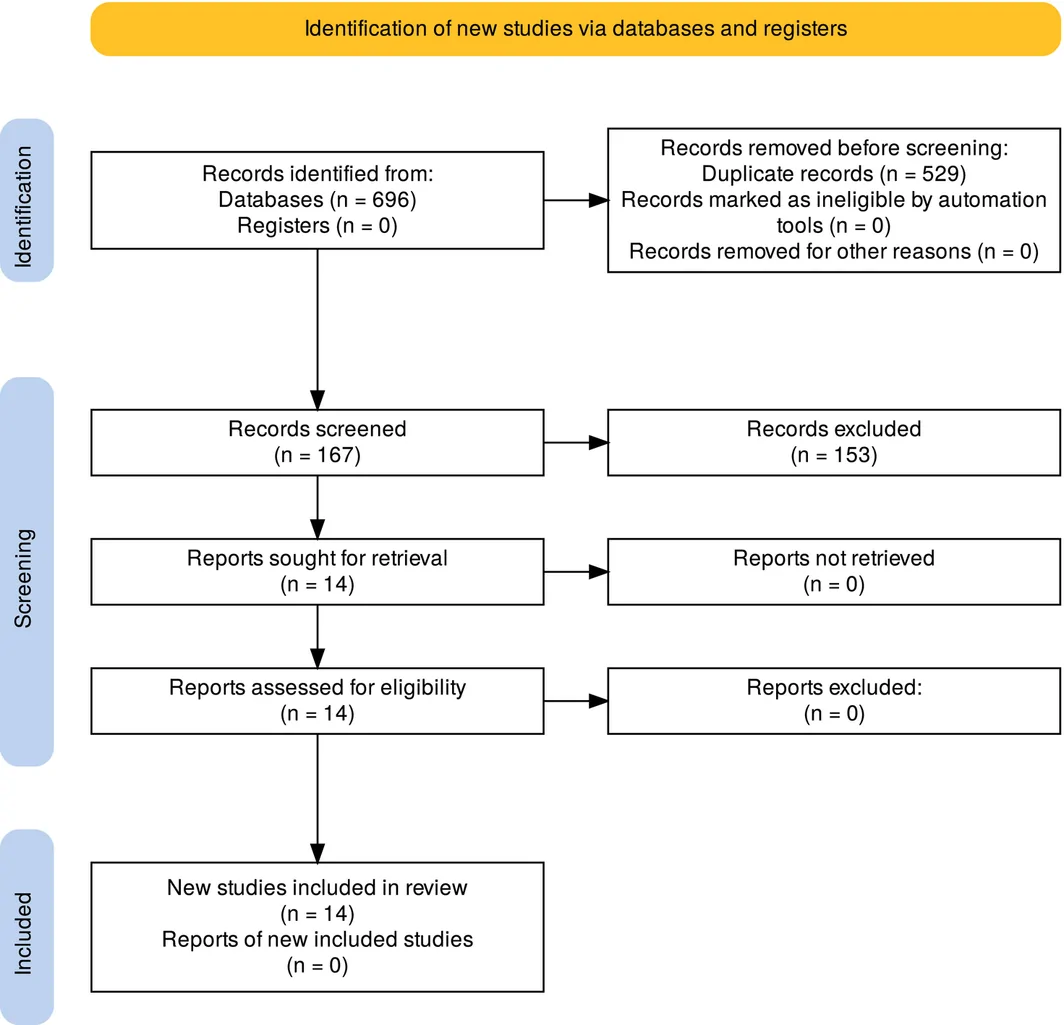
Prisma flowchart of study selection.

### 3.2 Study Characteristics

The characteristics of the 14 studies that met the inclusion criteria are presented in Table 1. The included trials were conducted between 2007 [31] and 2026 [30] and included a total of 776 subjects [22, 26, 27, 28, 29, 30, 31, 32, 33, 34, 35, 36, 37, 38], primarily middle‐aged adults. Sample sizes ranged from 5 [31] to 165 [28]. Five studies were randomized controlled trials: Cunha et al. [26], Holmer et al. [27], Hansen et al. [28], Chirapongsathorn et al. [29] and Farrell et al. [30]. The remaining nine studies were single‐arm prospective or cohort interventions: Tendler et al. [31], Luukkonen et al. [22], D'Abbondanza et al. [32], De Nucci et al. [33], Rinaldi et al. [34], Sila et al. [35], Haley et al. [36], Saslow et al. [37] and Barrea et al. [38].

The intervention periods ranged from 6 days [22] to 6 months. Hansen et al. [28] conducted a 6‐month intervention followed by a 3‐month follow‐up. The majority of participants had liver steatosis at baseline, assessed by ultrasonography, CAP, MRI‐based techniques, liver biopsy, or validated non‐invasive indices. The interventions consisted of various ketogenic diets, as described in Table S1. When control groups were included, participants received alternative dietary interventions, namely standard low‐calorie diet (LC) for weight loss, a 5:2 intermittent fasting diet (5:2 IF), a high‐carbohydrate low‐fat diet (HCLF), or a Mediterranean diet (MD) [26, 27, 28, 30].

### 3.3 Results of Individual Studies

A summary of the results of the individual studies is shown in Table 2. All articles considered a significance level of *p* < 0.05. An overview of the direction of effects across outcome domains, stratified by study design, is provided in Figure 2.

**TABLE 2:** Summary of the effects of ketogenic dietary interventions on anthropometric, hepatic, metabolic and enzymatic outcomes in adults with metabolic dysfunction–associated steatotic liver disease.

Outcome domain	Outcome	Direction of change	Range across studies	Summary interpretation
Anthropometric outcomes	Body weight	↓	−2.6 to −12.8 kg	Clinically meaningful weight reduction
	Body mass index	↓	−1.0 to −4.0 kg/m^2^	Improved adiposity profile
	Waist circumference	↓	−2.6 to −9.6 cm	Reduced central adiposity
	Fat mass	↓	−1.5 to −8.4 kg	Lowered total body fat
Hepatic outcomes	Liver fat (MRI‐PDFF/MRI‐LFF/MRS/IHTG)	↓	−3.1% to −77%	Substantial decrease in intrahepatic fat, although findings were heterogeneous across assessment methods
	CAP (FibroScan)	↓	−43.5 to −82.5 dB/m	Marked reduction in steatosis
	Liver stiffness	↓/↔	−0.2 to −1.1 kPa	Modest, inconsistent improvement
Metabolic outcomes	Fasting glucose	↓	−5.49 to −25.4 mg/dL	Improved glycaemic control
	Insulin	↓	−4.04 to −10.22 μU/mL	Lower fasting insulin
	Insulin resistance	↓	−1.16 to −2.9	Enhanced insulin sensitivity
	Triglycerides	↓	−17.7 to −53.5 mg/dL	Lower circulating lipids
	Total cholesterol	↓	−13.58 to −35.4 mg/dL	Moderate improvement
	LDL‐cholesterol	↓/↔/↑	−29.2 to +8.9 mg/dL	Variable response across studies
	HDL‐cholesterol	↓/↔/↑	−6.7 to +4.42 mg/dL	Variable response across studies
Liver enzymes	Alanine aminotransferase	↓	−3.3 to −38.7 U/L	Reduced hepatocellular injury
	Aspartate aminotransferase	↓	−4.4 to −15.3 U/L	Moderate improvement
	Gamma‐glutamyl transferase	↓	−3.85 to −25.0 U/L	Consistent reduction

*Note:* Ranges represent the overall direction and magnitude of change observed across both randomized controlled trials and prospective intervention studies. Detailed study‐level findings for each design are reported in Tables S2a–S2c. Direction of change: ↓ decrease; ↑ increase; ↔ no significant change.

**FIGURE 2:**
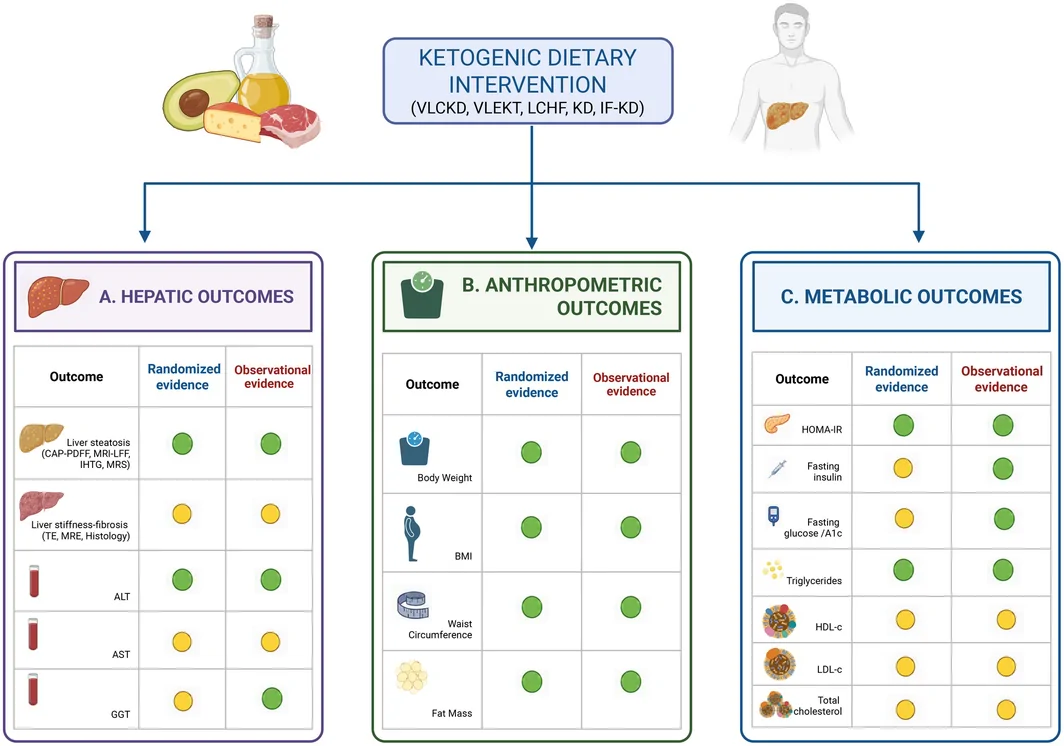
Overview of the direction of effects of ketogenic dietary interventions on hepatic, anthropometric and metabolic outcomes, stratified by study design (randomized vs. observational evidence). Created in https://BioRender.com.

### 3.4 Anthropometric Variables

Anthropometric outcomes were reported in all included studies. Overall, KD interventions were consistently associated with reductions in body weight (BW), body mass index (BMI), waist circumference (WC) and fat mass (FM), although the magnitude of change varied according to study design, intervention duration and dietary protocol. A summary of these results is presented in Table S2a.

Among the five RCT, clinically meaningful weight loss was observed across all KD intervention groups. Body weight reductions ranged from −5.5 to −12.0 kg in the intervention groups, compared with smaller reductions in control groups receiving standard dietary advice, Mediterranean diet, high‐carbohydrate low‐fat diet, or intermittent fasting regimens. Significant decreases in BMI (up to −4.0 kg/m^2^), waist circumference (up to −9.6 cm) and fat mass (up to −8.4 kg) were also reported, generally favouring ketogenic interventions over control diets [26, 27, 28, 29, 30].

Observational studies also reported similar findings. Weight loss ranged from −2.6 to −12.8 kg, BMI reductions from −1.0 to −4.0 kg/m^2^, and waist circumference reductions from −2.6 to −8.8 cm. Improvements in body composition were consistently observed, with reductions in fat mass ranging from −1.5 to −7.5 kg or equivalent percentage changes. The largest weight loss was reported by Tendler et al. [31], while substantial reductions in adiposity‐related measures were also observed in the other observational studies [22, 32, 33, 34, 35, 36, 37, 38]. Across both study designs, the direction of effect was remarkably consistent, suggesting that ketogenic dietary interventions effectively reduce overall and central adiposity in adults with MASLD.

### 3.5 Hepatic Outcomes

All the included studies evaluated the effect of ketogenic diets on hepatic outcomes (Table S2b). Hepatic outcomes were assessed using a broad range of methodologies, including MRI‐derived techniques (PDFF, MRI‐LFF and IHTG), CAP, ultrasonography, histology and validated non‐invasive indices, resulting in substantial methodological heterogeneity across studies. Overall, KD interventions were generally associated with improvements in hepatic steatosis, although findings were not uniformly significant across studies; by contrast, findings regarding liver stiffness (LS) were more heterogeneous.

Among the five RCTs, KD interventions generally favoured reductions in liver fat content, although the magnitude and statistical significance of these effects varied across studies and assessment methods. Improvements were documented using MRI‐derived techniques, CAP and histological evaluation, with reductions ranging from −4.77% in PDFF to a 77% relative reduction in MRI‐LFF. Reductions in CAP ranging from −43.5 to −82.5 dB/m were observed. By comparison, effects on liver stiffness were more heterogeneous, with significant improvements observed in some interventions, whereas others showed little or no change [26, 27, 28, 29, 30].

The nine prospective and observational studies reported similar findings. Specifically, significant reductions in hepatic steatosis were observed using MRI‐based techniques, CAP, ultrasonography, histology and non‐invasive indices. Improvements ranged from a 31% reduction in IHTG after only 6 days of intervention to marked reductions in CAP and significant improvements in histological or ultrasonographic measures of steatosis. In contrast, changes in liver stiffness were generally modest and frequently non‐significant, with only one study reporting a significant improvement [22, 31, 32, 33, 34, 35, 36, 37, 38]. Across both study designs, a similar direction of effect was observed, indicating that KD interventions improve hepatic steatosis. However, evidence regarding fibrosis‐related outcomes and liver stiffness remains limited and less consistent than for liver fat reduction.

### 3.6 Metabolic Outcomes

Metabolic outcomes were reported in most included studies and are summarized in Table S2c. Parameters evaluated included fasting glucose, glycated haemoglobin (HbA1c), insulin, HOMA‐IR, triglycerides (TG), total cholesterol (TC), low‐density lipoprotein cholesterol (LDL‐c) and high‐density lipoprotein cholesterol (HDL‐c). Overall, ketogenic dietary interventions were associated with improvements in glycaemic control, insulin sensitivity and circulating lipid concentrations, although the magnitude of change varied across studies and outcome measures. However, lipid‐related findings, particularly for LDL‐c and HDL‐c, were more heterogeneous and less consistent than those observed for glycaemic markers and triglycerides [26, 27, 28, 29, 30].

The nine prospective and observational studies reported similar findings. Significant improvements were observed in fasting glucose, insulin, HOMA‐IR, triglycerides, total cholesterol and LDL‐c, while HDL‐c showed a more variable response. Reductions reached −13% for fasting glucose, −53% for insulin and −57% for HOMA‐IR in short‐term ketogenic interventions, whereas longer VLCKD interventions consistently improved lipid profiles, with triglyceride reductions ranging from approximately −22 to −29 mg/dL and total cholesterol reductions ranging from −27 to −35 mg/dL. Significant reductions in HbA1c were also reported in several studies, supporting the beneficial effects of ketogenic interventions on glycaemic control and insulin sensitivity [22, 31, 32, 33, 34, 35, 36, 37, 38].

Across both study designs, a similar direction of effect was observed, indicating that ketogenic dietary interventions improve glycaemic regulation and insulin sensitivity while generally exerting favourable effects on lipid metabolism. However, changes in LDL‐c and HDL‐c were more heterogeneous than those observed for glucose metabolism and triglycerides.

### 3.7 Liver Enzymes

Liver enzyme outcomes were reported in most included studies and are summarized in Table S2c. Alanine aminotransferase (ALT), aspartate aminotransferase (AST) and gamma‐glutamyl transferase (GGT) were the most frequently assessed markers. Overall, ketogenic dietary interventions were associated with reductions in liver enzyme concentrations, suggesting improvements in hepatocellular injury and liver‐related biochemical parameters, although the magnitude of change varied across studies.

Among the five RCTs, ketogenic interventions generally resulted in favourable changes in liver enzymes. Reductions in ALT ranged from −15.4 to −26.0 U/L, while GGT reductions ranged from −4.5 to −25.0 U/L. Effects on AST were less consistent, with significant improvements observed in some trials but not others. Overall, liver enzyme improvements tended to parallel reductions in hepatic steatosis and body weight [26, 27, 28, 29, 30].

The nine prospective and observational studies reported similar findings. Significant reductions in GGT were observed across several studies, ranging from −3.85 to −15.89 U/L, whereas improvements in ALT were generally modest but significant when present. Changes in AST were less consistently reported and frequently failed to reach statistical significance [22, 31, 32, 33, 34, 35, 36, 37, 38].

Across both study designs, GGT appeared to be the most responsive liver‐related biochemical marker, while ALT showed moderate improvement and AST demonstrated the greatest variability. Overall, the observed reductions in liver enzymes were consistent with the improvements reported for hepatic steatosis and metabolic health.

### 3.8 Quality Assessment

All studies included in this review were evaluated using the CASP checklist for RCTs (Table S3) and the ROBINS‐I tool for non‐randomized interventional studies (Table S4). The five randomized controlled trials included in this review—Cunha et al. [26], Holmer et al. [27], Hansen et al. [28], Chirapongsathorn et al. [29] and Farrell et al. [30]—were evaluated following the CASP criteria, and the assessment revealed several methodological strengths and weaknesses across studies. All randomized trials reported participant allocation to intervention groups. Nevertheless, methodological concerns remained. The CASP assessment identified an unclear research question in Chirapongsathorn et al. [29]. In addition, incomplete participant follow‐up was common across studies. Hansen et al. [28] was the only trial that accounted for all participants at study completion, while reporting was unclear in Cunha et al. [26] and incomplete in Holmer et al. [27], Chirapongsathorn et al. [29] and Farrell et al. [30].

Blinding procedures were generally limited across the RCTs. None of the studies blinded participants or investigators, indicating a potential risk of performance bias. Outcome assessor blinding was reported only in Cunha et al. [26] and Farrell et al. [30] whereas Holmer et al. [27], Chirapongsathorn et al. [29] and Hansen et al. [28] did not report blinding of outcome assessors. Regarding baseline comparability, Holmer et al. [27], Hansen et al. [28] and Farrell et al. [30] provided adequate information showing that intervention groups were similar at the start of the study, whereas Cunha et al. [26] and Chirapongsathorn et al. [29], did not include sufficient data to confirm initial group equivalence.

The assessment also considered whether study groups received similar levels of care aside from the dietary intervention. Holmer et al. [27] showed inconsistencies in co‐interventions across arms, which may have influenced outcomes, while Cunha et al. [26] and Chirapongsathorn et al. [29] appeared to provide comparable care across groups. All three studies reported the effects of their dietary interventions comprehensively.

When examining statistical reporting, only Holmer et al. [27], Chirapongsathorn et al. [29], Hansen et al. [28] and Farrell et al. [30] provided adequate precision measures for the estimated effects, while Cunha et al. [26] did not report sufficient information to assess statistical precision. For all trials, it was not possible to determine whether the benefits of the ketogenic or comparator interventions outweighed potential harms and costs due to limited reporting. Furthermore, applicability to local or broader populations was uncertain for Holmer et al. [27], Chirapongsathorn et al. [29], Hansen et al. [28] and Farrell et al. [30], whereas the intervention proposed by Cunha et al. [26] was judged not directly applicable in different contexts.

Finally, none of the RCTs provided enough evidence to conclude whether the experimental intervention would offer greater value than existing treatments for individuals in similar clinical settings. Taken together, the overall CASP assessment indicates that although the five RCTs contribute valuable evidence, several methodological limitations—particularly concerning blinding, follow‐up completeness, baseline comparability and applicability—restrict the certainty and generalizability of their findings.

Analysing the nine non‐randomized interventional studies—Tendler et al. [31], Luukkonen et al. [22], D'Abbondanza et al. [32], De Nucci et al. [33], Rinaldi et al. [34], Sila et al. [35], Haley et al. [36], Saslow et al. [37] and Barrea et al. [38]—a serious risk of bias due to confounding was identified in several of them, particularly in the studies by Luukkonen et al. [22], Tendler et al. [31], Saslow et al. [37] and Barrea et al. [38] reflecting the absence of randomization and the likelihood that unmeasured clinical or behavioural differences between participants influenced the outcomes. Most studies showed a low risk of bias in participant selection, with the exception of D'Abbondanza et al. [32] and Barrea et al. [38], which showed a moderate risk, indicating that inclusion procedures were appropriate and consistently applied across samples. A moderate risk of misclassification of interventions was identified in Rinaldi et al. [34] and De Nucci et al. [33], mainly due to limited detail regarding adherence to the dietary protocol or the precise macronutrient composition of the ketogenic diet. Deviation from intended interventions was considered a moderate risk only in Tendler et al. [31] and Barrea et al. [38], while information in other studies was either insufficient or suggested low concern. Missing data represented an issue in some cases: De Nucci et al. [33] had a moderate risk, whereas the study by Haley et al. [36] exhibited a serious risk due to substantial loss to follow‐up over the two‐week intervention period. Regarding outcome measurement, all the studies demonstrated a moderate risk due to variability in assessment tools and incomplete methodological details. In the case of Luukkonen et al. [22], insufficient information was available to adequately evaluate measurement bias. Finally, selective reporting posed a serious risk in four studies—Luukkonen et al. [22], Tendler et al. [31], Rinaldi et al. [34] and De Nucci et al. [33]—primarily because not all prespecified outcomes were fully reported or because analytic procedures lacked clarity. Overall, most non‐randomized studies were judged to have a moderate risk of bias, with the exception of D'Abbondanza et al. [32], Sila et al. [35], which showed an overall low risk, reflecting inherent limitations such as the absence of control groups, variable intervention durations and heterogeneity in outcome assessment methods. These factors should be carefully considered when interpreting the effect estimates of ketogenic dietary interventions on MASLD.

## 4 Discussion

This systematic review evaluates the effect of a KD on a wide spectrum of hepatic and extra‐hepatic outcomes in patients with MASLD, including liver‐specific severity indices like liver enzymes, anthropometric measures, glycaemic indices and lipid profile. Evidence from five RCTs and nine prospective studies was evaluated. A total of 776 adults with MASLD were involved. The study results suggest that the KD may exert favourable effects across several clinically relevant domains, particularly in reducing liver steatosis and improving anthropometric and metabolic parameters. However, these findings should be interpreted with caution. Evidence is still limited and heterogeneous across studies. In addition, the observed improvements may be driven by energy restriction and weight loss rather than by the specific macronutrient composition of ketogenic diets. Notably, the degree of prescribed caloric restriction also varied considerably across RCTs: Cunha et al. [26] and Farrell et al. [30] employed markedly hypocaloric VLCKD/VLEKT protocols, whereas Hansen et al. [28] and, at least in its LCHF arm, Holmer et al. [27] did not prescribe caloric restriction. Even so, in Hansen et al. [28] the ad libitum LCHF arm still achieved greater weight loss than the comparator, illustrating how difficult it is to disentangle the metabolic effects of macronutrient composition from those of spontaneous caloric deficit even by design.

These clinical findings are, at least in part, biologically plausible: evidence from preclinical studies [41, 42] suggests that ketogenic diets may influence liver metabolism by reducing fat accumulation and modulating inflammatory processes. While these mechanisms help to explain the observed clinical effects, their direct applicability to human disease remains uncertain.

Beyond biological plausibility, however, the strength and reliability of the reported effects varied considerably across studies, reflecting several distinct sources of methodological heterogeneity. A first source relates to study population and design: sample size, intervention duration and the criteria used to diagnose and quantify MASLD differed substantially across the included studies. A second source concerns methodological rigour: although RCTs generally demonstrated higher methodological rigour than observational studies, several lacked blinding and differed substantially in intervention characteristics, follow‐up duration and outcome assessment methods, resulting in only moderate certainty of evidence overall. Outcome assessment also varied substantially across studies. Liver fat and disease severity were measured using MRI, CAP, ultrasonography, histology and surrogate indices such as FLI. These differences limit direct comparisons across studies and require cautious interpretation of the findings. Reassuringly, none of the included studies exhibited a critically high risk of bias, although this methodological variability still requires cautious interpretation of the overall body of evidence.

Several limitations should therefore be acknowledged. First, substantial heterogeneity existed in study populations, including differences in sample size, age, sex distribution, geographical setting, baseline disease severity and diagnostic criteria used to define hepatic steatosis. Such variability may limit the comparability of findings across studies. Second, intervention duration varied considerably, ranging from 6 days to 6 months, which limits the ability to extrapolate short‐term findings to long‐term MASLD progression. Third, dietary intake and adherence were frequently assessed using self‐reported measures, introducing the potential for recall and reporting bias. Fourth, sex‐specific effects remain poorly understood. Only one included study [32] specifically investigated sex‐related differences in response to intervention, reporting greater reductions in body weight and gamma‐glutamyl transferase among men than among pre‐menopausal women, while no significant sex difference was observed for the degree of hepatic steatosis itself. Consequently, current evidence is insufficient to determine whether sex modifies the effects of ketogenic diets in MASLD, highlighting the need for adequately powered sex‐stratified studies.

A second set of limitations concerns the assessed outcomes. Most reported measures were surrogate markers, such as liver enzymes and/or imaging‐based measures of steatosis and fibrosis. Evidence on clinically meaningful endpoints, including fibrosis progression or histological improvement, remains limited. Interpretation of the findings from Farrell et al. [30] requires particular caution, as low‐dose semaglutide was introduced after the initial 12‐week ketogenic intervention during the maintenance phase, meaning longer‐term outcomes cannot be attributed exclusively to the KD intervention itself.

Finally, inconsistent reporting across studies further constrains interpretation: data on physical activity, concomitant medication and dietary adherence were also inconsistently reported across studies and, when available, were assessed using heterogeneous methods, potentially contributing to residual confounding. This is consistent with the risk‐of‐bias assessment which showed moderate concerns related to confounding, misclassification, missing data and selective reporting, which may affect the reliability of the observed outcomes.

Importantly, our review combined evidence from both RCTs and prospective observational studies. While RCTs offer the strongest design for causal inference, observational cohorts are inherently more susceptible to residual confounding and baseline differences between participants who choose to follow a KD and those who did not. These systematic differences—related to motivation, disease severity, concurrent lifestyle changes, or unmeasured health behaviours—may partially account for the improvements observed in non‐randomized settings.

Accordingly, the effects derived from observational cohorts should be interpreted with greater caution, as the strongest evidence was derived from the randomized controlled trials. As a result, the overall body of evidence must be viewed in light of the differing methodological robustness of the included study designs.

Despite these weaknesses, this review offers several strengths. To the best of our knowledge, evidence on KD in MASLD has previously been synthesized primarily through narrative reviews, which rarely use systematic search methods or formal risk‐of‐bias assessment. By adopting a PRISMA‐guided approach and established quality tools, this review provides a structured and transparent overview of the available clinical evidence across a broad spectrum of hepatic and metabolic outcomes, allowing for a holistic analysis of KD's therapeutic potential. Furthermore, the absence of publication date restrictions ensured comprehensive literature retrieval and minimized selection bias.

Regarding applicability, the findings of this review suggest that KD protocols across studies were generally feasible, accessible and well‐tolerated. Patient‐specific factors such as genetics, habitual diet and baseline metabolic status should nevertheless be considered when individualizing dietary treatment.

## 5 Conclusion

KD appears to improve liver steatosis and several anthropometric and metabolic outcomes in adults with MASLD based on the available reviewed literature. Current evidence suggests that KD may be an effective dietary strategy for improving liver steatosis and metabolic outcomes in MASLD. Nevertheless, the available evidence is not yet strong enough to support its universal use as a standard treatment. Further randomized controlled trials are required.

## Author Contributions

**Patrizia Palumbo:** data curation; writing – original draft; writing – review and editing. **Harini Krishnapillai:** conceptualization; literature search; methodology; data curation; investigation; study selection; writing – review and editing. **Fleur de Vreeze:** conceptualization; literature search; methodology; data curation; investigation; study selection; writing – review and editing. **Elena Buzzetti:** supervision; writing – review and editing. **Ger H. Koek:** supervision; writing – review and editing.

## Funding

The authors have nothing to report.

## Conflicts of Interest

The authors declare no conflicts of interest.

## Supporting information


**Table S1:** Extended classification of dietary patterns.
**Table S2a:** Anthropometric outcomes following ketogenic or low‐carbohydrate interventions.
**Table S2b:** MASLD severity indices: changes in liver steatosis and liver stiffness across interventions.
**Table S2c:** Study results—metabolic outcomes: glycaemic, lipid and liver enzyme changes.
**Table S3:** Risk of bias assessment for randomized studies using the CASP tool.
**Table S4:** Risk of bias assessment for non‐randomized studies using the ROBINS‐I tool (NI = no information).
**Data S5:** Full search strategy.

**Data S1:** PRISMA reporting checklist.

## Data Availability

All data extracted for this review are contained within the article and its Supporting Information S1.
