# Supplementary material for: Ketogenic Diet Effects on Liver Health in Metabolic Dysfunction–Associated Steatotic Liver Disease: A Systematic Review
Source: Liver Int. 2026 Nov 13;46(12):e70942. doi: 10.1111/liv.70942 (PMC13763151; doi:10.1111/liv.70942)
Supplement: Supplementary file 1 [file LIV-46-0-s001.docx]

# **PRISMA Reporting Checklist**

| **Section/topic** | **#** | **Checklist item** | **Reported on page #** |
| --- | --- | --- | --- |
| **TITLE** | | |  |
| Title | 1 | Identify the report as a systematic review, a meta-analysis, or both. | Manuscript,  Title Page, page 1 |
| **ABSTRACT** | | |  |
| Structured summary | 2 | Provide a structured summary covering, as applicable: background; objectives; data sources; eligibility criteria; participants and interventions; appraisal and synthesis methods; results; limitations; conclusions and implications; and the systematic review registration number. | Manuscript,  Pages 6-7 |
| **INTRODUCTION** | | |  |
| Rationale | 3 | Explain the rationale for the review in the context of existing knowledge. | Manuscript,  pages 8-9 |
| Objectives | 4 | State the review questions explicitly in terms of participants, interventions, comparisons, outcomes, and study design (PICOS). | Manuscript,  page 9 |
| **METHODS** | | |  |
| Protocol and registration | 5 | State whether a review protocol exists, where it can be accessed (for example, via a web address), and, if available, its registration details and number. | N.A. |
| Eligibility criteria | 6 | Define the study characteristics (for example, PICOS and follow-up duration) and report characteristics (such as years covered, language, and publication status) used to determine eligibility, and explain the rationale. | Manuscript,  page 10 |
| Information sources | 7 | List all information sources used in the search, including database coverage dates and contacts with study authors, and report the date of the most recent search. | Manuscript,  pages 10-11 |
| Search | 8 | Provide the complete electronic search strategy for at least one database, including all limits, so that the search can be reproduced. | Manuscript,  page 11, Supplementary material 5 |
| Study selection | 9 | Describe the study-selection process, including screening, eligibility assessment, inclusion in the systematic review, and, where applicable, inclusion in the meta-analysis. | Manuscript,  page 11 |
| Data collection process | 10 | Describe how data were extracted from reports, including any piloted forms, independent duplicate extraction, and procedures used to obtain or confirm data with investigators. | Manuscript,  page 12 |
| Data items | 11 | List and define all variables for which data were sought, including PICOS elements and funding sources, and describe any assumptions or simplifications. | Manuscript,  page 12 |
| Risk of bias in individual studies | 12 | Describe how risk of bias was assessed for individual studies, whether at the study or outcome level, and how the assessment informed any data synthesis. | Manuscript,  page 12 |
| Summary measures | 13 | State the principal summary measures, such as risk ratios or differences in means. | Manuscript,  pages 12-13 |
| Synthesis of results | 14 | Describe how data were handled and study results combined, including the consistency measure used for each meta-analysis (for example, I^2^). | N.A. |

| **Section/topic** | **#** | **Checklist item** | **Reported on page #** |
| --- | --- | --- | --- |
| Risk of bias across studies | 15 | Describe any assessment of bias that could affect the cumulative evidence, such as publication bias or selective reporting within studies. | N.A. |
| Additional analyses | 16 | Describe any additional analyses performed, such as sensitivity analyses, subgroup analyses, or meta-regression, and identify those specified in advance. | N.A. |
| **RESULTS** | | |  |
| Study selection | 17 | Report the numbers of studies screened, assessed for eligibility, and included in the review; give reasons for exclusions at each stage, preferably in a flow diagram. | Manuscript,  page 13, Figure 1 |
| Study characteristics | 18 | For each study, present the characteristics for which data were extracted, such as sample size, PICOS elements, and follow-up period, and provide the relevant citations. | Manuscript,  pages 13-14,  Table 1 |
| Risk of bias within studies | 19 | Present risk-of-bias data for each study and, where available, assessments at the outcome level (see Item 12). | Manuscript,  pages 18-20, Supplementary Tables 3-4 |
| Results of individual studies | 20 | For every outcome considered—benefit or harm—report for each study: (a) summary data for each intervention group; and (b) effect estimates with confidence intervals, preferably in a forest plot. | Manuscript,  pages 14-18, Supplementary Tables 2a-c |
| Synthesis of results | 21 | Report the results of each meta-analysis, including confidence intervals and measures of consistency. | N.A. |
| Risk of bias across studies | 22 | Report the results of any assessment of risk of bias across studies (see Item 15). | N.A. |
| Additional analysis | 23 | Report the results of any additional analyses, such as sensitivity analyses, subgroup analyses, or meta-regression (see Item 16). | N.A. |
| **DISCUSSION** | | |  |
| Summary of evidence | 24 | Summarize the main findings and the strength of evidence for each principal outcome, and explain their relevance to key groups such as healthcare providers, service users, and policy makers. | Manuscript,  pages 20-21 |
| Limitations | 25 | Discuss limitations at the study and outcome levels, including risk of bias, and at the review level, such as incomplete retrieval of research or reporting bias. | Manuscript,  pages 22-23 |
| Conclusions | 26 | Interpret the results in the context of the wider evidence and explain their implications for future research. | Manuscript,  page 24 |
| **FUNDING** | | |  |
| Funding | 27 | Report all sources of funding and other support for the systematic review, such as the provision of data, and describe the funders’ role. | Manuscript, page 4 |
