# Supplementary material for: Ketogenic Diet Effects on Liver Health in Metabolic Dysfunction–Associated Steatotic Liver Disease: A Systematic Review
Source: Liver Int. 2026 Nov 13;46(12):e70942. doi: 10.1111/liv.70942 (PMC13763151; doi:10.1111/liv.70942)
Supplement: Supplementary file 2 [file LIV-46-0-s002.docx]

**Supplementary materials**

*Supplementary Table 1: Extended Classification of Dietary Patterns*

|  | Kcal/day | CHO/day | Fat/day | Ketosis |
| --- | --- | --- | --- | --- |
| High-fat KD (HFKD) | Unrestricted | < 20-50g | Unrestricted | Yes |
| Very low-calorie KD (VLCKD)  Very low-energy Ketogenic Therapy (VLEKT)* | <800 kcal | <20-50g | Low | Yes |
| Very low-calorie diet (VLCD) | < 800 kcal | >20-50g | Low | Usually not |
| Low-carbohydrate diet (LCD) | Variable | <130g | Low | No |

*Adapted from Watanabe et al.^21^*

**VLEKT (Very Low-Energy Ketogenic Therapy) is the updated terminology proposed for very low-calorie ketogenic diets (VLCKD) and was considered equivalent to VLCKD in the present review*

*Supplementary Table 2a: Anthropometric Outcomes Following Ketogenic or Low‑Carbohydrate Interventions*

| **Study  (Author, Year)** | **BW**  **(kg)** | **BMI  (kg/m²)** | **WC (cm)** | **FM**  **(kg)** |
| --- | --- | --- | --- | --- |
| **Cunha et al., 2020^35^** | VLCKD: −9.7 ± 3.9*** LC: −1.67 ± 2.2** | VLCKD: −3.56 ± 1.3*** LC: −0.71 ± 0.96** | VLCKD: −8.00 ± 3.9*** LC: −3.0 ± 2.9** | NA |
| **Holmer et al., 2021^32^** | 5:2 IF: −7.4*** LCHF: −7.3***  SoC: −2.5*** | 5:2 IF: −2.4*** LCHF: −2.5***  SoC: −0.9*** | **WHR**  5:2 IF: −0.03*** LCHF: −0.05**  SoC: −0.02** | NA |
| **Hansen et al., 2023 ^38^** | LCHF: −5.5***;  HCLF: −1.7†;  Between-group Δ −3.8 kg* | NA | LCHF: −5.1*;  HCLF: −2.3*;  Between-group Δ −2.8 cm* | LCHF: −1.30%;  HCLF: −0.27%;  Between-group Δ −1.04%* |
| **Chirapongsathorn et al., 2025^27^** | KD: −6.1***  Control: −2.14*** | NS | KD: −9.6*  Control: −4.22* | KD: −4.8**  Control: −1.42** |
| **Farrell et al., 2026 ^33^** | VLED: −12.0*** MD: −3.5* | VLED: −4.0*** MD: −1.2* | NA | VLED: −8.4*** MD: −2.9* |
| **Tendler et al., 2007^26^** | −12.8* | NA | NA | NA |
| **Luukkonen et al., 2020^22^** | −2.8** | −1.0*** | −2.6** | −1.5*** |
| **D'Abbondanza et al., 2020 ^39^** | −10.0*** | −4.0*** | −8.0*** | FM%: −3.0*** |
| **De Nucci et al., 2023^31^** | NA | −3.0*** | −8.2*** | −6.8*** |
| **Rinaldi et al., 2023^34^** | NA | −2.95** | −8.03** | −7.49** |
| **Sila et al., 2024^28^** | NA | −3.1*** | −8.82*** | −6.65*** |
| **Haley et al., 2025^36^** | −2.6*** (overall) | NA | NA | NA |
| **Saslow et al., 2025^37^** | −10.7* (overall)  −11.7* (adherent) | −3.7* (overall)  −4.1* (adherent) | NA | NA |
| **Barrea et al., 2025 ^40^** | −7.12 kg*** | −2.66 kg/m²*** | −6.61 cm*** | −6.31 kg*** |

* = p < 0.05; ** = p < 0.01; *** = p < 0.001; † = NS (not significant); NA = Not assessed; Values expressed as change from baseline (Δ) unless otherwise stated. Units and statistical reporting as per original studies. BW= body weight; BMI= body mass index; WC= waist circumference; WHR= waist to hip ratio; FM= fat mass; VLCKD= Very Low-Calorie Ketogenic Diet; LC= Low Carb diet; 5:2 IF=5:2 Intermittent Fasting; LCHF= Low Carb High Fat Diet; SoC=Standard of Care; KD= Ketogenic Diet, VLEKT= Very Low Energy Ketogenic Therapy; VLED= Very Low Energy Diet.

For studies reporting multiple follow-up time points, outcomes were extracted at the end of the ketogenic dietary intervention. In Farrell et al., 2026, 12-week data were reported because semaglutide was introduced during the subsequent maintenance phase, with the exception of the histological outcome (Supplementary Table S2b), which was only available at week 24 and is flagged accordingly.

*Supplementary Table 2b: MASLD severity indices: Changes in Liver Steatosis and Liver Stiffness Across Interventions*

| **Study  (Author, Year)** | **Diagnostic Method** | **Hepatic Steatosis**  **(%-dB/m)** | **Liver Stiffness**  **(kPa)** |
| --- | --- | --- | --- |
| **Cunha et al., 2020^35^** | PDFF (MRI) | VLCKD: −4.77 ± 4.26%***  LC: −0.79 ± 1.76%*** | NS |
| **Holmer et al., 2021^32^** | CAP (FibroScan®, MRE) | 5:2 IF: −63.8 dB/m***  LCHF: −61.9 dB/m***  SoC: −20.2 dB/m† | 5:2 IF: −1.8***  LCHF: −0.3†  SoC: −1.5** |
| **Hansen et al., 2023 ^38^** | Histology | NS† | NA |
| **Chirapongsathorn et al., 2025^27^** | CAP (FibroScan®, MRE) | KD: −43.5 dB/m†  Control: −13.3 dB/m† | NS† |
| **Farrell et al., 2026 ^33^** | MRI-LFF, CAP, Histology | MRI-LFF: −77%***  Absolute LFF: −18% CAP: −82.5 dB/m*  Histological steatosis (week 24, post-semaglutide ‡): −1.5 stage** | −1.1* |
| **Tendler et al., 2007^26^** | Histology | Improvement in 4/5 paired biopsies* | NA |
| **Luukkonen et al., 2020^22^** | IHTG (H‑MRS) | −31%*** | NS† |
| **D'Abbondanza et al., 2020 ^39^** | Ultrasonography | Significant reduction in steatosis grade*** | NA |
| **De Nucci et al., 2023^31^** | CAP (FibroScan®, MRE) | −57 dB/m*** | −0.2* |
| **Rinaldi et al., 2023^34^** | CAP (FibroScan®, MRE) | −43.61 dB/m** | NS† |
| **Sila et al., 2024^28^** | CAP (FibroScan®, MRE) | −47 dB/m*** | NS† |
| **Haley et al., 2025^36^** | PDFF (MRI) | −3.1%** | NA |
| **Saslow et al., 2025^37^** | PDFF (MRI) | −33.2% (overall) †  −53.1% (adherent)* | NS† |
| **Barrea et al., 2025 ^40^** | FLI | FLI: −14.03*** | NA |

* = p < 0.05; ** = p < 0.01; *** = p < 0.001; † = NS (not significant); NA = Not assessed; Values expressed as change from baseline (Δ) unless otherwise stated. Units and statistical reporting as per original studies. PDFF= Proton Density Fat Fraction; MRI= Magnetic Resonance Imaging; CAP= Controlled Attenuation Parameter; MRE= Magnetic Resonance Elastography; IHTG= Intrahepatic Triglycerides; H‑MRS= Proton Magnetic Resonance Spectroscopy; FLI= Fatty Liver Index; MRI-LFF = Magnetic Resonance Imaging Liver Fat Fraction.

‡ In Farrell et al., 2026, the follow-up liver biopsy was performed only at week 24 (after the introduction of low-dose semaglutide during the maintenance phase); no histological data are available at week 12. This is the only outcome from this study reported at 24 rather than 12 weeks; it should therefore be interpreted with caution, as it may partly reflect the effect of semaglutide rather than the ketogenic intervention alone.

*Supplementary Table 2c: Study results – Metabolic Outcomes: Glycemic, Lipid, and Liver Enzyme Changes*

| **Study  (Author, Year)** | **TG**  **(mg/dl)** | **TC**  **(mg/dl-mmol/l)** | **LDL-c (mg/dl- mmol/l)** | **HDL-c**  **(mg/dl - mmol/l)** | **Glucose**  **(mg/dl)** | **HbA1c**  **(%-mmol/mol)** | **Insulin**  **(μU/ml)** | **HOMA-IR** | **ALT**  **(U/L)** | **AST**  **(U/L)** | **GGT**  **(U/L)** |
| --- | --- | --- | --- | --- | --- | --- | --- | --- | --- | --- | --- |
| **Cunha et al., 2020^35^** | NS† | VLCKD: −13.58*  mg/dl  LC: −13.88**  mg/dl | NS† | NS† | NS† | −0.11%* | NS† | NS† | NS | VLCKD −3.95* | NS |
| **Holmer et al., 2021^32^** | 5:2 IF: −35.43*  LCHF: −17.71*  SoC: −17.71† | 5:2 IF: −0.50***  mmol/l  LCHF: +0.2†  SoC: −0.07† | 5:2 IF: −0.40*** mmol/l  LCHF: ↑ trend†  Soc: −0.05† | 5:2 IF: +0.05†  mmol/l  LCHF: +0.10*  Soc: +0.05† | NA | 5:2 IF: −4.8*** mmol/mol  LCHF: −3.2***  mmol/mol  SoC: −1.1† mmol/mol | NA | 5:2 IF: −3.2***  LCHF: −2.9**  SoC: −2.4† | 5:2 IF −17.6***  LCHF −17.6*  SoC −11.8** | 5:2 IF −6.0†  LCHF −4.9†  SoC −9.1* | NA |
| **Hansen et al., 2023 ^38^** | LCHF: −32.7† HCLF: −17.7† | NA | LCHF: +8.9*** mg/dl HCLF:  −5.8†  mg/dl  Between-group Δ: +14.3***  mg/dl | LCHF: +3.9† mg/dl | LCHF: −25.4*** HCLF: −10.6*** Between-group Δ: −14.8*** | LCHF: −0.88%*** HCLF: −0.29%* Between-group Δ: −0.59%*** | NA | LCHF: −2.38* HCLF: −0.25† Between-group Δ: −2.13* | NA | NA | NA |
| **Chirapongsathorn et al., 2025^27^** | KD: −53.5*  Control:  -14.5* | NS† | NS† | KD: −5.08*  mg/dl  Control: +1.2*  mg/dl | NS† | NA | NA | NA | KD: −15.4†  Control: −1.2† | KD: −6.5*  Control: +0.2* | NA |
| **Farrell et al., 2026 ^33^** | −26.57* | NA | 0† | +0.05†  mmol/l | NA | −0.4%* | NA | −1.8* | −26* | NA | −25* |
| **Tendler et al., 2007^26^** | NS† | NS† | NS† | −15.9%*  TC/HDL | NA | NA | NS† | NA | NS† | NA | NA |
| **Luukkonen et al., 2020^22^** | −25%** | NS† | NS† | NS† | −13%** | NA | −53%** | −57%** | NS† | NS† | −10* |
| **D'Abbondanza et al., 2020 ^39^** | NA | NA | NA | NA | NA | −1.4*** | NA | −2.6* | NS† | NS† | −12.0*** |
| **De Nucci et al., 2023^31^** | −22.76*** | −27.54*** mg/dl | −20.51***  mg/dl | −5.72*** mg/dl | −7.32*** | −0.22%*** | −6.69*** | −1.82*** | −5.27*** | NS† | −8.47*** |
| **Rinaldi et al., 2023^34^** | −26.40** | −35.40** mg/dl | −27.58** mg/dl | −6.66** mg/dl | −8.93** | NA | −4.04** | −1.16** | −3.33** | NS† | −3.85** |
| **Sila et al., 2024^28^** | −24.80*** | −27.09***  mg/dl | −20.98***  mg/dl | −6.28*** mg/dl | −5.49*** | −0.20%*** | −6.91*** | −1.71*** | −6.14* | NS† | −8.67*** |
| **Haley et al., 2025^36^** | NA | NA | NA | NA | NA | NA | NA | NA | NA | NA | NA |
| **Saslow et al., 2025^37^** | −16.00* (overall) | NA | NS † | NS † | −10.00* (overall) | −3.89* (overall) | −7.87* (overall) | −2.34* (overall) | NS † | NS † | NA |
| **Barrea et al., 2025 ^40^** | −29.24*** | −30.63***  mg/dl | −29.20***  mg/dl | +4.42***  mg/dl | −16.01*** | NA | NA | NA | −8.67*** | −6.20*** | −15.89*** |

* = p < 0.05; ** = p < 0.01; *** = p < 0.001; † = NS (not significant); NA = Not assessed; Values expressed as change from baseline (Δ) unless otherwise stated. Units and statistical reporting as per original studies. VLCKD= Very Low-Calorie Ketogenic Diet; LC= Low Carb diet; 5:2 IF=5:2 Intermittent Fasting; LCHF= Low Carb High Fat Diet; SoC=Standard of Care; KD= Ketogenic Diet, VLEKT= Very Low Energy Ketogenic Therapy; VLED= Very Low Energy Diet.

*Supplementary Table 3: Risk of bias assessment for randomized studies using the CASP tool*

| Assessment items | Study 1 Cunha^35^ | Study 2 Holmer^32^ | Study 3  Hansen ^38^ | Study 4 Chirapongsathorn^27^ | **Study 5 Farrell^33^** |
| --- | --- | --- | --- | --- | --- |
| Did the study address a clearly formulated research question? | Yes | Yes | Yes | No | Yes |
| Was the assignment of participants to interventions randomized? | Yes | Yes | Yes | Yes | Yes |
| Were all participants accounted for at its conclusion? | Can’t tell | No | Yes | No | No |
| Were the participants ‘blind’ to the intervention they were given? | No | No | No | No | No |
| Were the investigators ‘blind’ to the intervention they were giving to participants? | No | No | No | No | No |
| Were the people assessing/analyzing outcome/s ‘blinded’? | Yes | No | No | No | Yes |
| Were the study groups similar at the start of the RCT? | Can’t tell | Yes | Yes | Yes | Yes |
| Apart from the experimental intervention, did each study group receive similar level of care? | Yes | No | Yes | Yes | Yes |
| Were the effects of the intervention reported comprehensively? | Yes | Yes | Yes | Yes | Yes |
| Was the precision of the estimate of the intervention or treatment effect reported? | No | Yes | Yes | Yes | Yes |
| Do the benefits of the experimental intervention outweigh the harms and costs? | Can’t tell | Can’t tell | Can’t tell | Can’t tell | Can’t tell |
| Can the results be applied to your local population/in your context? | No | Can’t tell | Can’t tell | Can’t tell | Can’t tell |
| Would the experimental intervention provide greater value to the people in your care than any of the existing interventions? | Can’ tell | Can’t tell | Can’t tell | Can’t tell | Can’t tell |

*Supplementary Table 4: Risk of bias assessment for non-randomized studies using the ROBINS-I tool (NI = no information)*

| Assessment items | Study 6  Luukkonen^22^ | Study 7 **D'Abbondanza^39^** | Study 8  De Nucci^31^ | Study 9  Rinaldi^34^ | Study 10  Tendler^26^ | Study 11  Sila^28^ | Study 12 Haley^36^ | Study 13 Saslow^37^ | Study 14  Barrea^40^ |
| --- | --- | --- | --- | --- | --- | --- | --- | --- | --- |
| Bias due to confounding | Serious | Moderate | Moderate | Moderate | Serious | Moderate | Moderate | Serious | Serious |
| Bias in selection of participants into the study | Low | Moderate | Low | Low | Low | Low | Low | Low | Moderate |
| Bias in classification of interventions | Low | Low | Moderate | Moderate | Low | Low | Low | Low | Low |
| Bias due to deviations from intended interventions | Low | Low | NI | Low | Moderate | Low | Low | Low | Moderate |
| Bias due to missing data | Low | Low | Moderate | Low | Low | Low | Serious | Low | Low |
| Bias in measurement of outcomes | NI | Moderate | Moderate | Moderate | Moderate | Moderate | Moderate | Moderate | Moderate |
| Bias in selection of the reported result | Serious | Low | Serious | Serious | Serious | Moderate | Moderate | Moderate | Low |
| Overall bias | Moderate | Low | Moderate | Moderate | Moderate | Low | Moderate | Moderate | Moderate |

# *Supplementary Material 5: Full search*

((((((Diet, Ketogenic[MeSH Terms]) OR (Low-carb diet)) OR (Ketogenic Diet)) OR (Diets, Ketogenic)) OR (Ketogenic Diets)) OR (Low-carbohydrate high-fat diet)) OR (Very-low-carbohydrate diet) AND ((((((Ketogenic Diet[MeSH Terms]) OR (Low-carb diet)) OR (Ketogenic Diet)) OR (Diets, Ketogenic)) OR (Ketogenic Diets)) OR (Low-carbohydrate high-fat diet)) OR (Very-low-carbohydrate diet) AND (((((((((Fatty Liver[MeSH Terms]) OR (NAFLD)) OR (fatty liver)) OR (non-alcoholic fatty liver disease)) OR (Nonalcoholic steatohepatitis)) OR (Hepatic steatosis)) OR (Liver fat accumulation)) OR (Nonalcoholic liver disease)) OR (MASLD)) OR (metabolic dysfunction-associated steatotic liver disease) AND (((((((((Non-alcoholic Fatty Liver Disease[MeSH Terms]) OR (NAFLD)) OR (fatty liver)) OR (non-alcoholic fatty liver disease)) OR (Nonalcoholic steatohepatitis)) OR (Hepatic steatosis)) OR (Liver fat accumulation)) OR (Nonalcoholic liver disease)) OR (metabolic dysfunction-associated steatotic liver disease)) OR (MASLD) AND ((((((((Liver Diseases[MeSH Terms]) OR (NAFLD)) OR (fatty liver)) OR (non-alcoholic fatty liver disease)) OR (Nonalcoholic steatohepatitis)) OR (Hepatic steatosis)) OR (Liver fat accumulation)) OR (Nonalcoholic liver disease)) OR (MASLD)) OR (metabolic dysfunction-associated steatotic liver disease)

*Updated search strategy performed during revision*

To address reviewer comments and ensure inclusion of studies adopting the newer nomenclature for fatty liver disease, an updated search was conducted in PubMed, Scopus and Web of Science using the following additional queries:

("ketogenic diet"[Title/Abstract]) AND ("MASLD"[Title/Abstract])

("ketogenic diet"[Title/Abstract]) AND ("MAFLD"[Title/Abstract])

("ketogenic diet"[Title/Abstract]) AND ("NAFLD"[Title/Abstract])
